# Immune response following a two-dose schedule of bivalent HPV vaccination among girls and boys

**DOI:** 10.3389/fimmu.2024.1327770

**Published:** 2024-01-26

**Authors:** Marit Middeldorp, Janneke W. Duijster, Jan van de Kassteele, Fiona R.M. van der Klis, Hester E. de Melker

**Affiliations:** ^1^ Centre for Infectious Disease Control, National Institute for Public Health and the Environment, Bilthoven, Netherlands; ^2^ Department of Epidemiology and Data Science, Amsterdam University Medical Centre (UMC), Location VU University medical centre (VUmc), Amsterdam, Netherlands

**Keywords:** human papillomavirus, HPV, vaccine induced antibody response, GMC - geometric mean concentration, bivalent HPV vaccine, gender neutral vaccination

## Abstract

**Background:**

This longitudinal cohort study describes the kinetics in antibody levels after two doses of the bivalent human papillomavirus (HPV) vaccine in girls (birth cohort 2001) vaccinated in the routine Dutch vaccination program at 12 years of age, up to 7.5 years post-vaccination. Also, the antibody response one month post-vaccination of the first cohort of boys (birth cohort 2012, vaccinated at 10 years of age) eligible for HPV vaccination in the Netherlands is presented.

**Method:**

Blood samples and questionnaire data were collected of girls and boys. HPV type-specific antibody concentrations (LU/mL) against HPV16/18/31/33/45/52/58 were assessed using a validated virus-like particle (VLP) multiplex immunoassay. For girls, antibody decays over time were modelled using the modified power-law decay model and the exponential decay model.

**Results:**

The Geometric Mean Concentrations (GMCs) remained higher for HPV16/18 than for HPV types 31, 33, 45, 52, and 58 among girls up to 7.5 years post-vaccination. The antibody levels of HPV16 and HPV18 reached plateau values of 482 and 159 LU/mL, respectively. Mathematical modelling showed that the half-life values of HPV16/18 were 2.4- to 4.5-fold higher compared with the half-life values of the other HPV types. Among boys (aged 10 years), the GMC for HPV16 was significantly higher than among girls one month post-vaccination (aged 12 years).

**Conclusion:**

The GMCs of all HPV types declined over time, although the GMCs of HPV16/18 remained relatively high up to 7.5 years post-vaccination. The GMCs for HPV16/18 among boys were at least equally high as the GMCs among girls at one month post-vaccination. Further follow-up of the cohort of boys is needed to gain knowledge on long-term immune responses of young boys following bivalent HPV vaccination.

## Introduction

Infections with the human papillomavirus (HPV) are the most common sexually transmitted infections worldwide, with an estimated lifetime risk of getting infected of about 80% for people living in Western countries (1). Vaccination against HPV for the prevention of HPV-related cancers has been available since 2006. To date, three prophylactic HPV vaccines are licensed for the global market. Initially, HPV vaccines were licensed in a three-dose (3D) schedule (0, 1, and 6 months), which was later changed to a two-dose (2D) schedule (0 and 6 months) as the antibody responses in (9-16-year-old) girls who received a 2D schedule were found to be non-inferior to the responses in (15-26-year-old) women who received the 3D schedule (2, 3).

In the Netherlands, the bivalent HPV vaccine was introduced in the National Immunization Program (NIP) in 2010, by inviting girls for routine vaccination using a 3D schedule at 12 years of age. In 2014, the 3D schedule was replaced by the 2D schedule (4–6). In 2022, the age of vaccination was lowered to 10 years which is in accordance with Health Council’s recommendation, aiming to provide protection against HPV infections before onset of sexual activity (5–7). Moreover, since the same year, routine HPV vaccination in the NIP is being offered to 10-year old boys as well.

In 2014, a longitudinal study was initiated to examine the kinetics in antibody levels of vaccine-types HPV16/18 and other high-risk HPV-types (hrHPV-types 31/33/45/52/58) after vaccination with a 2D schedule of the bivalent vaccine in 12-year old girls (8). Results up to 24 months after vaccination showed that the antibody geometric mean concentrations (GMCs) declined over time for all HPV-types, but remained high for vaccine-types HPV16/18 (8). Long-term data on vaccine-induced anti-HPV antibody levels after a 2D schedule is limited, but data after vaccination with a 3D schedule is available for at least 12 years post-vaccination. In the Finnish Maternity Cohort in which females received the vaccine at 16–17 years of age, HPV16/18 antibody levels remained consistently stable above antibody levels induced by a natural infection (9). Moreover, sustained HPV6/11/16/18 antibody responses up to 14 years post-vaccination were reported among young Scandinavian women who received three doses of the quadrivalent HPV vaccine (at an age of 16-23 years) (10). The stability of vaccine-induced antibody levels contributes to long-term protection, which is pivotal considering the early age of vaccination (9). Further follow-up of the Dutch cohort can provide insight into antibody kinetics after two doses of the bivalent vaccine over time.

The expansion of the Dutch NIP to gender-neutral HPV vaccination was based on immunogenicity studies with boys (10-18 years old) who were vaccinated with a 3D schedule of the bivalent vaccine (11). Antibody levels in boys were found to be substantially higher than those in young women (15-25 years old). A comparison between boys and girls aged 10-14 years showed that the immune response of boys was non-inferior to that of girls. However, literature on the long-term immune response after vaccination with a 2D schedule of the bivalent vaccine among young boys is scarce. Therefore, a longitudinal study was initiated to examine the kinetics in antibody levels of vaccine types and five other hrHPV-types in boys vaccinated in the routine vaccination program at 10 years of age in 2022.

In this study, we describe the kinetics in antibody levels up to 7.5 years post-vaccination among girls vaccinated with two doses of the bivalent vaccine at 12 years of age. Furthermore, we present the antibody kinetics one month after the second dose among boys vaccinated with two doses of the bivalent vaccine at 10 years of age. To our knowledge, this is the first study evaluating the antibody kinetics from boys vaccinated in the routine vaccination program in the Netherlands.

## Materials and methods

### Study population and procedures

In 2014, this longitudinal cohort study among girls was initiated as described in detail by Schurink et al. (8). Briefly, the inclusion criteria comprised (a) being assigned female sex at birth, (b) being born in 2001, (c) vaccinated with a 2D schedule of the bivalent HPV vaccine in 2014 within the routine program, and (d) having an interval of at least five months between the first and second dose of vaccination. Due to logistical reasons, only girls from a middle-sized municipality in the centre of the Netherlands (Amersfoort) were invited. A total of 198 girls were invited. Still, the selection of girls from this municipality was performed randomly from the Dutch national vaccination registry Praeventis (12). Participating girls were asked to complete an online questionnaire and to provide a blood sample at one month, six months, and thereafter annually up to 7.5 years after the second dose of vaccination.

In 2022, a cohort study of boys born in 2012 was initiated with a similar design as the study in girls. In total, 1163 boys who were vaccinated with a 2D schedule (having at least five months interval between the two doses) of the bivalent HPV vaccine in 2022, were invited to participate. To ensure an interval of one month between the second dose of vaccination and sampling, only boys who received their first dose around mid-April were eligible for random selection from the Dutch vaccination registry Praeventis (12). Participating boys were requested to complete an online questionnaire and to draw a self-sample of finger-prick blood one month post-vaccination with the second dose. For both cohorts, a gift card was provided after each round blood sample has been sent in.

Participants were requested to provide a blood sample by either venepuncture at a selected sampling location (using a serum tube of VACUETTE, Greiner Bio-one, North Carolina, USA) (only applicable for the girls cohort) or by means of a self-sample of finger prick blood (Whatman 903 Protein Saver Card, GE Healthcare, Cardiff, United Kingdom). The serological analysis of the protein saver cards has been previously described (13, 14). A previous validation pilot (unpublished) found a strong correlation between the dried blood spot from the protein saver cards and conventional serum measurements for all HPV types (R^2^, 0.94–0.99; correlation coefficients, 0.91–1.07). The interchangeable use of venepuncture or finger prick blood was validated, resulting in a correction either on the raw lab data (first three rounds) or simultaneously during plate read-out and lab analysis (from 2016 onwards) (8). Due to the restrictions in place during the COVID-19 pandemic, the method of blood collection changed. From 2020 onwards, exclusively the possibility to draw a self-sample of finger-prick blood at home was offered (using the Microvette^®^ 300 Serum, Sarstedt, Germany).

### Serological measurements

A virus-like particle (VLP)–based multiplex immunoassay was used to quantify type-specific HPV antibodies to types 16, 18, 31, 33, 45, 52, and 58. To analyse antibodies post-vaccination, we used HPV VLPs produced by GlaxoSmithKline (GSK) and Merck & Co. VLPs were linked to 7 distinct color-coded fluorescent microspheres, and the multiplex immunoassay was performed as previously described (14, 15). In brief, samples were serially diluted up to 1/20,000 and incubated with the VLP-coupled microspheres. Sera were incubated with the VLP-coupled microspheres. HPV-specific IgG antibodies were detected using R-phycoerythrin (R-PE) conjugated goat anti-human IgG (Jackson ImmunoResearch laboratories Inc, Westgrove, PA). Four ‘in-house’ control sera and an ‘in-house’ standard were used on each plate (15). The ‘in-house’ standard (IVIG, lot LE12H227AF, Baxter) was calibrated against reference serum of GSK for all the seven HPV types. HPV-specific antibodies were measured using the Bioplex system 200 or the Bioplex 3D system and analysed with Bioplex software (Bio-Rad Laboratories, Hercules, CA). For each analyte, median fluorescent intensity was converted to Luminex units per millilitre (LU/mL) (16) by using a twofold serial diluted reference standard (IVIG, lot LE12H227AF, Baxter) and interpolating the MFI data through a 5-parameter curve-fitting algorithm (8). For HPV16 and 18, arbitrary LU/mL can be converted to International Units per millilitre (IU/mL) by dividing LU/mL by 2.8 and 3.3, respectively (16).

### Statistical analysis

Sociodemographic characteristics among boys and girls were described per available study round. GMCs with corresponding 95% confidence intervals (CIs) for HPV types 16, 18, 31, 33, 45, 52, 58 were also determined per available study round. For the comparison of the GMCs between boys and girls at the first study, the Mann–Whitney test was used.

For participating girls, antibody decays after the second dose were modelled over time. In total 2,847 measurements were available, however, a total of 23 incorrect measurements originating from four different girls had to be excluded as correctness and the timing of sample collection was questionable for these samples.

The antibody decays over time were modelled using the modified power-law decay model as described by Fraser et al. (17). In this model, the antibody decay (because of the decline in active B-cells) is described by a power law. Simultaneously, the model accounts for memory B-cells, which enables the establishment of a long-term antibody plateau. On the log-scale, the antibody levels are described by the following equation: f(*t*) = *k* + log [*p* + (1 − *p*)(1 + *t*)^-^ *^a^*], where f(*t*) is the log-antibody level at time *t* (in years), *k* is the log-antibody level at *t* = 0, *p* is the relative level of antibodies produced in the long-term memory plateau (0 < *p* < 1), and *a* is a decay rate parameter (*a* > 0). The long-term plateau can be calculated by *b* = *k* + log(*p*).

We also considered an exponential decay model, as an alternative for the modified power-law decay model, to describe the log-antibody levels using the following equation: *f*(*t*) = *b* + (*k* - *b*) exp(-*a t*). All parameters have the same meaning as above. However, the decay rate parameters of the two models can not directly be compared due to the different mathematical representations of a decay process. We calculated the time in years for the log-antibody level to reach 50% and 25% of the initial value, relative to the long-term plateau level. Therefore, providing information about decay rates that can directly be compared between both models.

The models were formulated as mixed effects models, where all parameters were included as random effects, providing each participating girl her own antibody decay curve over time, while borrowing information from other girls simultaneously. In order to correct for sexual debut in the calculation of decay in GMCs over time, this variable was included as a fixed effect in both models (coded as 0 before, 1 after sexual debut, assuming the effect remains present after sexual debut).

The models were fitted in Stan. We ran four parallel Markov chain Monte Carlo- (MCMC) chains, each containing 12,500 samples. Mixing and convergence of the chains was assessed visually. Predicted antibody levels and their corresponding 95% CIs were calculated as individual concentrations (results not shown) and as GMCs (graphs). Parameter estimates and their corresponding 95% CIs are presented in tables. Both models were compared using the Bayesian leave-one-out (LOO) cross-validation information criterion (18). The LOO can be used to assess the model’s accuracy and compare different models based on their ability to generalize to new data. The model with the lowest LOO score is typically considered the best-performing model.

Statistical analyses were performed using SAS software package version 9.4 (SAS Institute Inc., Cary, NC, USA), R version 3.4.0^1^ and Stan version 2.21^2^.

### Ethical approval

This study adhered to the tenets of the Declaration of Helsinki and was approved by the Medical Ethics Committee of the VU University Medical Center in Amsterdam (2014.230). Informed consent of all participants was required before inclusion.

## Results

### Socio-demographic characteristics

A total of 56 of the 198 invited girls (28.3%) participated in this study. Of these girls, complete participation (i.e. both a questionnaire and blood sample provided) ranged between 53.6% and 98.2% during the nine study rounds. **Table 1** describes the socio-demographic characteristics per study round. The majority of the participating girls were of Dutch ethnicity. Over time, sexual activity increased to approximately 80% and almost all girls ever used contraception (93.9%). A maximum of two girls per study round used immunosuppressive medication, though, during most study rounds, none of the girls used immunosuppressive medication.

**Table 1:** Socio-demographic characteristics of the female participants (birth cohort 2001) per sampling moment.

Study round (time since vaccination)	R1 (Mo 1)	R2 (Mo 6)	R3 (Y 1.5)	R4 (Y 2.5)	R5 (Y 3.5)	R6 (Y 4.5)	R7 (Y 5.5)	R8 (Y 6.5)	R9 (Y 7.5)
Participation (n = 56) complete	55	54	52	45	47	47	30	32	31
*Blood only*	0	0	1	3	2	1	1	4	2
*Questionnaire only*	0	1	2	0	1	1	10	1	2
Calendar year	2014	2015	2016	2017	2018	2019	2020	2021	2022
Age (median, range)	13, 12-13	13, 13-14	14, 14-15	15, 15-16	16, 16-17	17, 17-18	18, 18-19	19, 19-20	20, 20-21
Ethnicity (% Dutch)	48 (81.4)	48 (81.4)	48 (81.4)	41 (91.1)	39 (90.8)	37 (90.3)	33 (91.7)	27 (93.1)	23 (95.8)
Current educational level^*^									
*High*	29 (52.7)	29 (52.7)	29 (53.7)	22 (48.9)	25 (52.1)	25 (56.8)	18 (51.4)	20 (69.0)	22 (75.9)
*Middle*	18 (32.7)	20 (36.4)	21 (38.9)	18 (40.0)	19 (39.6)	19 (43.2)	17 (48.6)	9 (31.0)	7 (24.1)
*Low*	8 (14.6)	6 (10.9)	4 (7.4)	5 (11.1)	4 (8.3)	0 (0.0)	0 (0.0)	0 (0.0)	0 (0.0)
Ever had sex (% yes)	0 (0.0)	0 (0.0)	0 (0.0)	5 (11.1)	12 (25.0)	18 (38.3)	24 (60.0)	23 (69.7)	26 (78.8)
Age sexual debut among sexually active participants (median, range)	N/A	N/A	N/A	15, 14-15	15, 15-16	16, 14-17	16, 14-19	16, 13-19	17, 15-20
Ever used contraception (% yes)	4 (7.3)	5 (9.0)	9 (16.7)	17 (37.8)	26 (53.1)	32 (66.7)	30 (76.9)	29 (82.9)	29 (93.6)
Underlying illness (% yes)	2 (3.6)	3 (5.5)	4 (7.4)	2 (4.4)	5 (10.4)	5 (10.4)	2 (5.0)	2 (6.1)	3 (9.1)
Immunosuppressive medication (% yes)	1 (1.9)	0 (0.0)	0 (0.0)	1 (2.3)	0 (0.0)	2 (4.3)	0 (0.0)	0 (0.0)	2 (6.3)
Had menarche (% yes)	39 (70.9)	46 (83.6)	51 (94.4)	45 (100)	48 (100)	48 (100)	40 (100)	33 (100)	33 (100)

Mo, Month; Y, Year; N/A, Not applicable.

^*^ Low = primary or lower general vocational secondary education; Middle = intermediate vocational secondary education; High = higher vocational/general secondary education, (pre)university education.

In 2022, a total of 1463 boys (birth cohort 2012) were invited to participate in the study. Of these boys, 81 boys were included in the study of which 55 boys completed participation (serum sample and questionnaire), 12 boys participated with only a serum sample, and 3 boys with only a questionnaire in the first study round. All boys were of Dutch ethnicity, and from the majority of the participating boys (79%) one or both of the parents had a high educational level. An underlying illness was reported among 7 boys (13%) of which all boys had a pulmonary disease (e.g. asthma). Of these boys, 3 boys additionally reported to have a skin condition (e.g. eczema) and 1 boy reported to have irritable bowel syndrome. In total, 1 boy (2%) ever used immunosuppressive medication.

### Decay in geometric mean concentrations over time among girls

**Figure 1:**
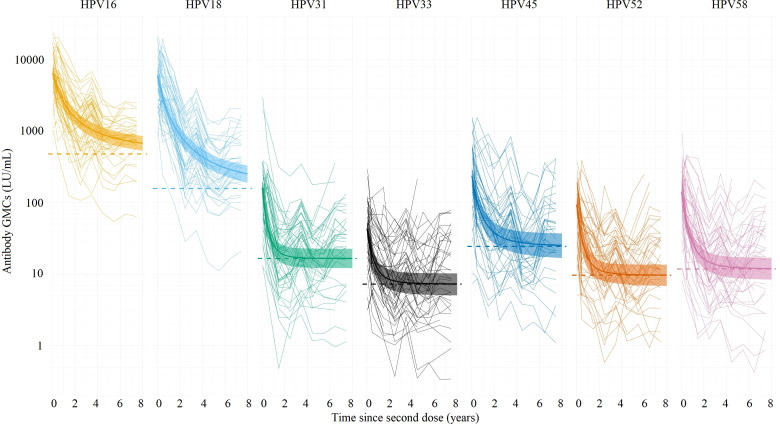
Estimated antibody geometric mean concentrations (GMCs; LU/mL) (thick lines) with their corresponding 95% Cis (ribbons) for HPV types 16/18/31/33/45/52/58 based on the power-law decay model, using individual antibody data measured up to 7.5 years (thin lines) after the second dose of vaccination among girls (birth cohort 2001). The dotted lines represent the estimated plateau GMCs using the power-law decay model (all types). LU, Luminex units.

**Figure 1** shows the GMCs (with corresponding 95% CIs) over time among girls adjusted for sexual debut, plotted over the individual data. **Table 2** shows the estimated parameter values for the power-law decay model.

**Table 2:** Model-based prediction of GMCs following 2vHPV vaccination.

Type	Initial GMC (LU/mL)	Plateau GMC (LU/mL)	50% of initial GMC w.r.t. plateau (years)	25% of initial GMC w.r.t. plateau (years)	GMC ratio sexual debut
HPV16	6606.4 (5439.0-8029.7)	481.9 (338.5-677.7)	1.7 (1.2-2.3)	4.2 (2.8-6.6)	1.0 (0.9-1.2)
HPV18	6000.0 (4792.8-7493.2	158.6 (100.8-235.0)	1.8 (1.4-2.4)	4.5 (3.3-6.3)	1.1 (0.9-1.3)
HPV31	162.9 (122.1-215.3)	16.6 (12.1-22.7)	0.4 (0.4-0.5)	0.8 (0.7-1.0)	1.4 (1.2-1.6)
HPV33	43.7 (33.6-58.0)	7.3 (5.0-10.2)	0.5 (0.4-0.7)	1.0 (0.7-1.4)	1.5 (1.3-1.8)
HPV45	239.4 (173.4-327.4)	24.5 (15.8-36.7)	0.7 (0.5-1.1)	1.6 (1.1-2.5)	1.2 (0.9-1.4)
HPV52	94.3 (71.3-124.6)	9.7 (6.8-13.5)	0.5 (0.3-0.8)	1.0 (0.6-1.7)	1.4 (1.2-1.7)
HPV58	142.3 (107.5-187.6)	11.8 (8.2-16.8)	0.6 (0.5-0.8)	1.3 (1.0-1.7)	1.4 (1.2-1.8)

GMC, geometric mean concentration; LU, Luminex units; w.r.t: with respect to.

GMCs predicted from the power law model, using antibody data measured up to 7.5 years after the second dose of vaccination among girls (birth cohort 2001).

All girls seroconverted for HPV types 16 and 18 after vaccination. Overall, the observed HPV16 and HPV18 antibody levels were still high 7.5 years after vaccination. The initial GMCs (i.e. the level just after the second dose) were 6606 LU/mL (HPV16) and 6000 LU/mL (HPV18), and were considerably higher compared to the other types. In the first 2.5 years post-vaccination, a marked decline in GMCs of HPV16/18 antibody levels was observed. Thereafter, a less steep decline was observed, and the antibody levels eventually reached a plateau. The plateau GMCs for HPV16 and HPV18 were estimated to be 482 and 159 LU/mL, respectively (**Table 2**). The plateaus for the other types were considerably lower. After 1.7 years (HPV16) and 1.8 years (HPV18), the GMC reached 50% of its initial antibody level (i.e. the level just after the second dose), with respect to the plateau value. After 4.2 years (HPV16) and 4.5 years (HPV18) the GMCs reached 25% of its initial level. For the other types, the GMC reached 50% and 25% of its initial antibody level with respect to the plateau value between 0.4 (HPV31) and 0.7 (HPV45) years, and between 0.8 (HPV31) and 1.6 (HPV45) years.

Sexual debut had no significant effect on the GMCs for HPV16, HPV18, and HPV45 over time among girls. For the other types, the GMC was between 1.4-1.5 times higher after sexual debut than before sexual debut.

Estimates for the exponential decay model are shown in **Supplementary Table 1** and **Supplementary Figures 1**-**5**. The results between the power-law decay model and the exponential decay model are quite comparable, with the exemption of the decay rates for HPV16 and HPV18. In the exponential model, the GMC’s reaches 25% of its initial antibody level with respect to the plateau value faster than in the power law model (for HPV16: 2.8 years *vs.* 4.2 years; for HPV18: 3.5 years *vs.* 4.5).

### Geometric mean concentrations boys *vs.* girls

**Figure 2:**
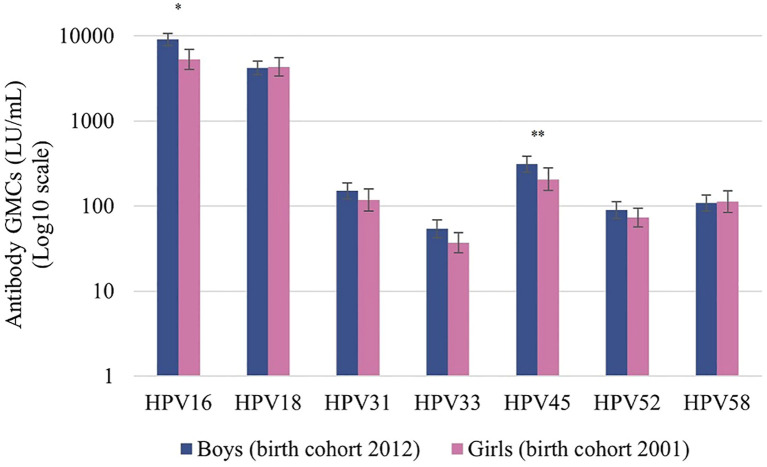
Antibody geometric mean concentrations (GMCs; LU/mL) of HPV types 16/18/31/33/45/52/58 at one month after the second dose of vaccination among boys (birth cohort 2012, vaccinated at 10 years) and girls (birth cohort 2001, vaccinated at 12 years). LU, Luminex units. Significance is displayed as * P <.001 and **P <.05.

All boys seroconverted for HPV types 16 and 18. HPV16 and HPV18 antibody levels among boys were high with GMCs of 9069 LU/mL and 4215 LU/mL, respectively. The GMC one month post-vaccination for HPV16 among boys (aged 10 years) was significantly higher than the GMC at the same interval among participating girls (aged 12 years) (**Figure 2**). The GMCs for HPV types 18, 31, 33, 45, 52, and 58 did not differ significantly between boys and girls one month post-vaccination.

## Discussion

This observational cohort study assessed the kinetics in GMCs against HPV-types 16, 18, 31, 33, 45, 52 and 58 in vaccinated girls (at 12 years of age) and boys (at 10 years of age) after a 2D schedule of the bivalent HPV16/18 vaccine. Among girls, results up to 7.5 years after the second dose of vaccination showed that GMCs remained highest for HPV16 and HPV18, compared to the GMC for HPV types 31, 33, 45, 52, and 58. Mathematical modelling of the long-term anti-HPV responses following vaccination, showed that the half-life values of HPV16 and 18 were respectively 4.5-fold and 2.4-fold higher than the half-life values of HPV types 31, 33, 45, 52, and 58. In addition, the quarter-life values were over almost 6 to 3 fold higher for HPV16 and 18 as compared to the other types. Among the cross-protective HPV types 31/33/45, the quarter-life value for HPV45 was 2 to 2.5-fold higher than for HPV31/33.

Declining GMCs for HPV16/18 were observed over the follow-up period, which is in line with the antibody responses of 12–15 year old girls that received a 3D schedule of the bivalent vaccine up to 7 years post-vaccination in the United Kingdom (19). Comparing the GMCs for HPV16/18 in our cohort with GMCs associated with naturally-acquired HPV16/18 infections as described previously (20), the GMCs remained consistently higher than the GMC of natural infections up to 7.5 years following vaccination. In a randomized trial, antibody responses against HPV16/18 36 months post-vaccination were 2.8-6.8-fold higher among 9-14-year old girls who received the bivalent HPV vaccine as compared to girls of the same age who received the quadrivalent HPV vaccine (21). This difference might be explained by the adjuvants used per vaccine as adjuvants play an important role in triggering the activation of an innate immunity pathway (22). We observed high GMCs against HPV16/18 in our study, however, translating these antibody concentrations to a degree of protection poses a difficulty, as there are no defined correlates of protection for HPV vaccination (23). In 2016, a prospective cohort study was initiated to estimate the vaccine effectiveness (VE) of bivalent vaccination against genital HPV infections after a 2D schedule in the Netherlands (24). Up to eight years post-vaccination, the protection against incident and persistent infection with HPV types 16/18 was high with VEs of 88% (95% CI: 69, 95) and 100% respectively (22). Hence, despite declining GMCs, the protection against genital HPV16/18 infections after two doses of the bivalent vaccine remains sufficiently high over time.

The antibody levels against HPV16/18 generated shortly post-vaccination were substantially higher than non-vaccine-type antibody levels and remained so during follow up (**Table 2**). Antibodies induced by the bivalent vaccine have the potential to cross-react with phylogenetically related HPV types 31, 33, 45, 52, and 58 (14, 25). These antibodies remain detectable for at least 12 years post-vaccination (26), yet, their levels decreased over time which might be explained by waning of cross-reactive antibody levels over a long follow-up period (19). Cross-protection against persistent HPV31/33/45 infections is observed after two doses of the bivalent vaccine (24), hence, it is likely that other immunological mechanisms are important. Earlier studies found that local immune responses (antibodies at the site of entry) should be considered. Type specific and cross-reactive HPV antibodies are important in the protection against disease but are only a part of the immunological compartment, as cellular-, innate- and mucosal immunity also play a role (27).

The power-law decay model was used to describe the GMCs over time. This model has been extensively used to model long-term HPV antibody dynamics (17, 28). As an alternative, the exponential model was used (**Supplementary Table 1**). Both models acceptably fitted the data and no statistically significant differences between the models were observed for all HPV types in terms of the LOO information criterion (**Supplementary Figures**). The time for the GMC to reach 25% of the initial value with respect to the plateau value was longer for the power-law decay model. This is consistent with the presumed antibody decay dynamics, where decay rates decrease over time (power-law decay) instead of being constant over time (exponential decay). This behaviour is better captured by the power-law decay model (17). Furthermore, in both models sexual debut had no significant effect on the GMCs for HPV16, HPV18, and HPV45 over time among girls (**Supplementary Figure 5**). Previously, no association was found between HPV16 antibody levels and sexual debut (29). However, an increasing number of lifetime sexual partners is associated with increasing HPV16 antibody levels (29, 30). Unfortunately, this information was not available in the current study.

GMCs are known to decrease fast after the first year after the second dose while thereafter the decline is less steep up to at least 3.5 years after the second dose (31). With regard to the individual plotted GMCs over time among girls (**Figure 1**), the GMCs seem to vary over time. A possible explanation for this finding is the variation within the multiplex immunoassay used for the current study. A previous study found a mean coefficient of variation (CV) for the intra-assay plate-to-plate variation and variation within a plate of 8% and 7% respectively, while the inter-assay variation, performed on different days, had a mean CV of 12% (32). In addition, this study covers sample collection and laboratory analysis over 9 years and conditions of essential materials have changed. Despite thorough bridging, we cannot exclude this might also have had an effect on the individual results. Furthermore, the percentage of participants who failed to provide a blood sample increased after the fourth study round (data not shown) which may slightly have biased the GMCs observed after this round.

Boys included in this study were the first cohort of boys eligible for two doses of the bivalent HPV vaccine in the Netherlands. The GMC for HPV16 was significantly higher among boys than among girls one month post-vaccination. This difference is possibly due to higher age at which girls were vaccinated in the routine vaccination program (at 12 years of age) than boys (at 10 years of age). This is in line with literature where post-vaccination antibody levels for both HPV types 16 and 18 were found to be up to 3-fold higher in boys aged 10 to 18 years than in women aged 15 to 25 years (11, 33, 34). In the present study, the age difference between boys and girls is smaller, nonetheless, age could have had an effect. Apart from this immunological difference, differences in the VLPs used between boys and girls could have had an effect on the observed antibody concentrations, however the serological method to determine antibody responses remained consistent. Significant differences between GMCs were not seen for types other than HPV16. For the nonavalent HPV vaccine, comparable GMCs for HPV types 16, 18, 31, 33, 45, 52, 58 between boys and girls 9-14 years of age were found one month after the second dose (35).

A strength of our study is the use of long-term follow-up data in the first cohort of girls who were vaccinated with two doses of the bivalent HPV vaccine during the routine immunization schedule in the Netherlands. Furthermore, we were able to compare the antibody kinetics in boys and girls one month after the second dose of vaccination. A key strength of our study is that we modelled the antibody decays over time using two different decay models; the modified power-law decay model and the exponential decay model. The long-term follow-up data collected in this study provides the opportunity to compare with accumulating immunogenicity data of a single-dose schedule, which is interesting given the strong interest in a single-dose HPV vaccination schedule globally. However, this study also has some limitations. This cohort comprises girls randomly selected from a single municipality due to logistical constraints. Despite the probability of some selection bias, it is anticipated that any influence of this potential bias on the results of the current study will be minimal. Another limitation is the variation within the multiplex immunoassay used for the current study which might had an effect to some extent on the individual antibody GMCs.

To conclude, high GMCs against vaccine-types HPV16/18 were observed up to 7.5 years of follow-up in girls vaccinated in the routine vaccination program with a 2D schedule with the bivalent vaccine. The GMC for HPV16/18 among boys vaccinated in the routine vaccination program with a 2D schedule at one month post-vaccination was at least as high as the GMCs among girls. This result is reassuring with regard to long-term immunogenicity of the 2D schedule against vaccine types among the first cohort of boys who were eligible for HPV vaccination in 2022. Information on long-term immune response after a 2D schedule of the bivalent vaccine among young boys is limited, so further follow-up of this cohort will provide insight into antibody concentrations over a longer period.

## Data availability statement

The raw data supporting the conclusions of this article will be made available by the authors, without undue reservation.

## Ethics statement

The studies involving humans were approved by The Medical Ethics Committee of Amsterdam University Medical Centers (UMC), the Netherlands. The studies were conducted in accordance with the local legislation and institutional requirements. Written informed consent for participation in this study was provided by the participants’ legal guardians/next of kin.

## Author contributions

MM: Conceptualization, Formal analysis, Writing – original draft. JD: Conceptualization, Writing – review & editing. JK: Formal analysis, Methodology, Visualization, Writing – review & editing. FK: Writing – review & editing. HM: Conceptualization, Writing – review & editing.

## Footnotes

1
https://www.R-project.org/

2
https://mc-stan.org/
