# Supplementary material for: Immune response following a two-dose schedule of bivalent HPV vaccination among girls and boys
Source: Front Immunol. 2024 Jan 26;15:1327770. doi: 10.3389/fimmu.2024.1327770 (PMC10853341; doi:10.3389/fimmu.2024.1327770)
Supplement: Supplementary file 1 [file DataSheet_1.pdf]

Supplementary Table 1. Model-based prediction of GMCs following 2vHPV vaccination. GMCs predicted from the exponential model, using antibody data measured up to 7.5 years after the second dose of vaccination among girls (birth cohort 2001).

Type	Initial GMC (LU/mL)	Plateau GMC (LU/mL)	50% of initial GMC w.r.t. plateau (years)	25% of initial GMC w.r.t. plateau (years)	GMC ratio sexual debut
HPV16	6270.6 (5053.5-7806.3)	648.4 (488.1-842.7)	1.4 (1.1-1.9)	2.8 (2.2-3.8)	1.1 (0.9-1.2)
HPV18	5502.3 (4353.2-6964.7)	210.1 (151.5-296.9)	1.7 (1.4-2.2)	3.5 (2.8-4.3)	1.1 (0.9-1.3)
HPV31	156.7 (111.7-222.9)	16.3 (12.5-21.4)	0.5 (0.4-0.6)	0.9 (0.8-1.1)	1.4 (1.2-1.6)
HPV33	44.1 (30.6-68.1)	6.9 (5.2-9.5)	0.5 (0.3-0.7)	1.0 (0.6-1.4)	1.5 (1.2-1.8)
HPV45	243.9 (168.6-349.6)	25.2 (17.8-34.8)	0.8 (0.5-1.1)	1.5 (1.0-2.2)	1.2 (1.0-1.5)
HPV52	98.0 (68.6-135.4)	10.5 (7.9-13.8)	0.5 (0.3-0.7)	0.9 (0.6-1.4)	1.4 (1.2-1.7)
HPV58	138.1 (93.2-197.3)	12.1 (8.5-16.3)	0.7 (0.6-0.8)	1.4 (1.1-1.7)	1.5 (1.2-1.7)

LU: Luminex units; w.r.t: with respect to

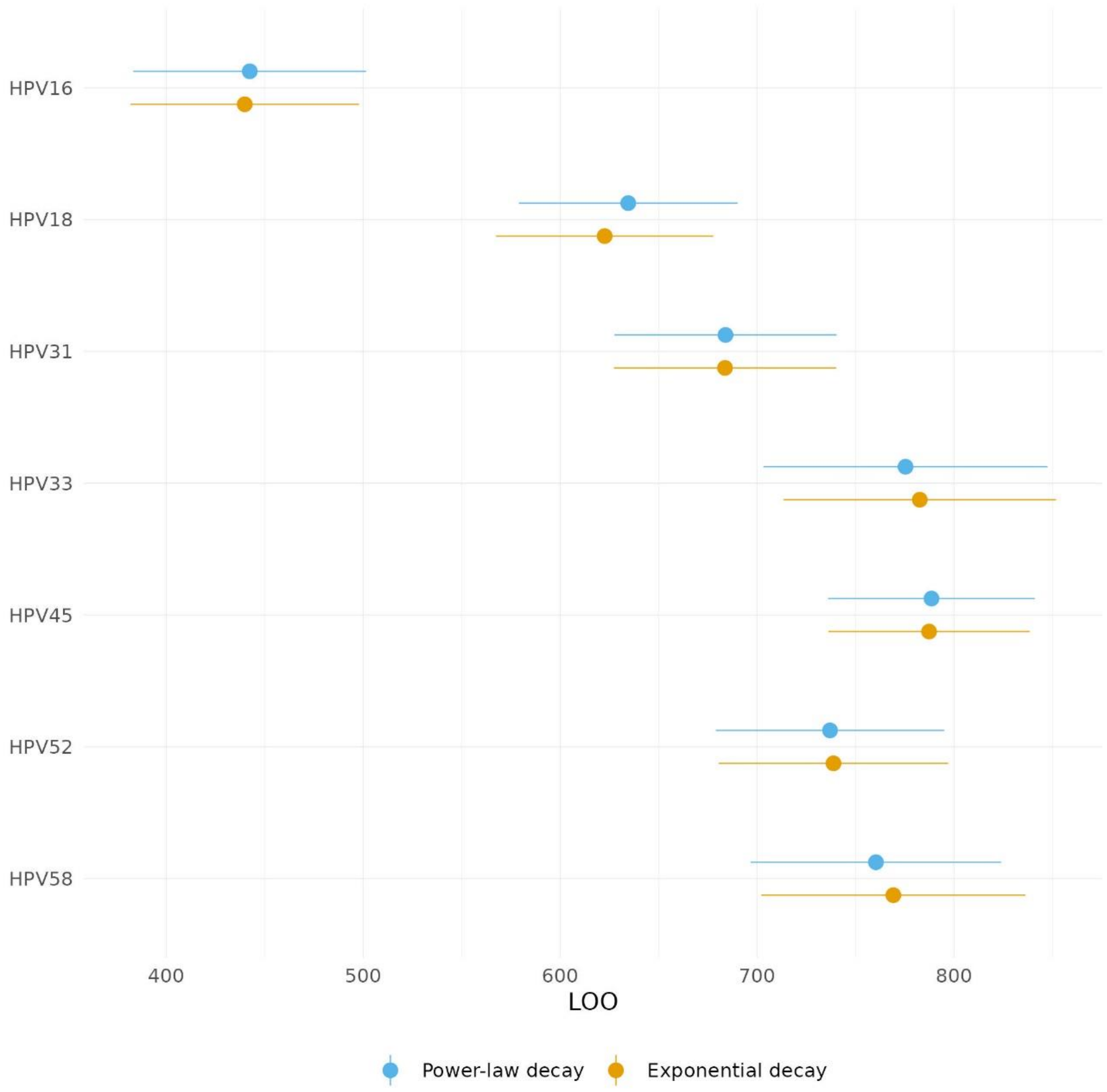

Supplementary Figure 2. Leave-one-out crossvalidation information criterion and their corresponding 95% CIs for each HPV type for the power-law decay model and expontential decay model.

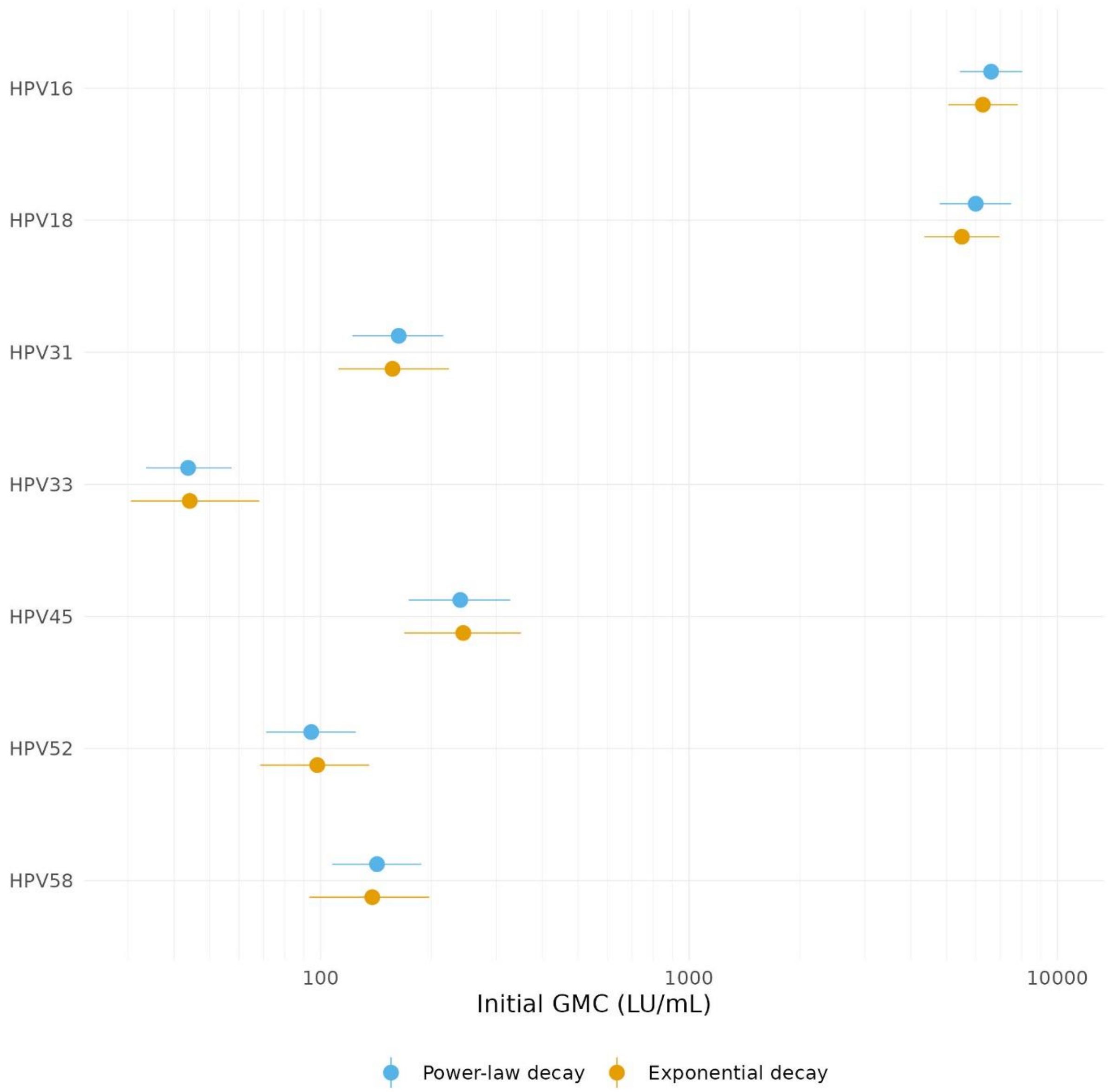

Supplementary Figure 2. Estimated initial GMC (LU/ml) and their corresponding 95% CIs for each HPV type just after the second dosis for the power-law decay model and expontential decay model.

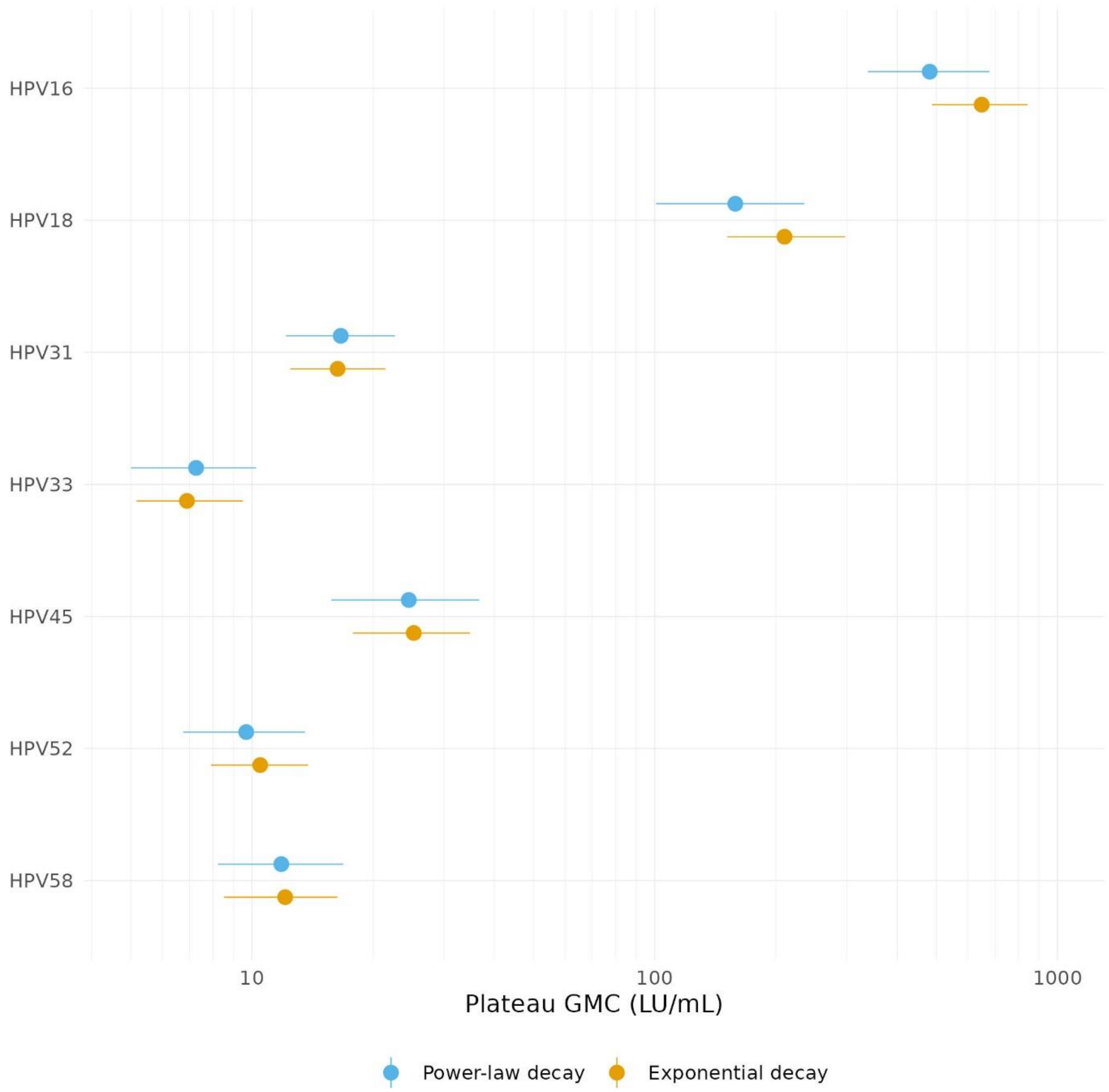

Supplementary Figure 3. Estimated plateau GMC (LU/ml) and their corresponding 95% CIs for each HPV type for the power-law decay model and expontential decay model.

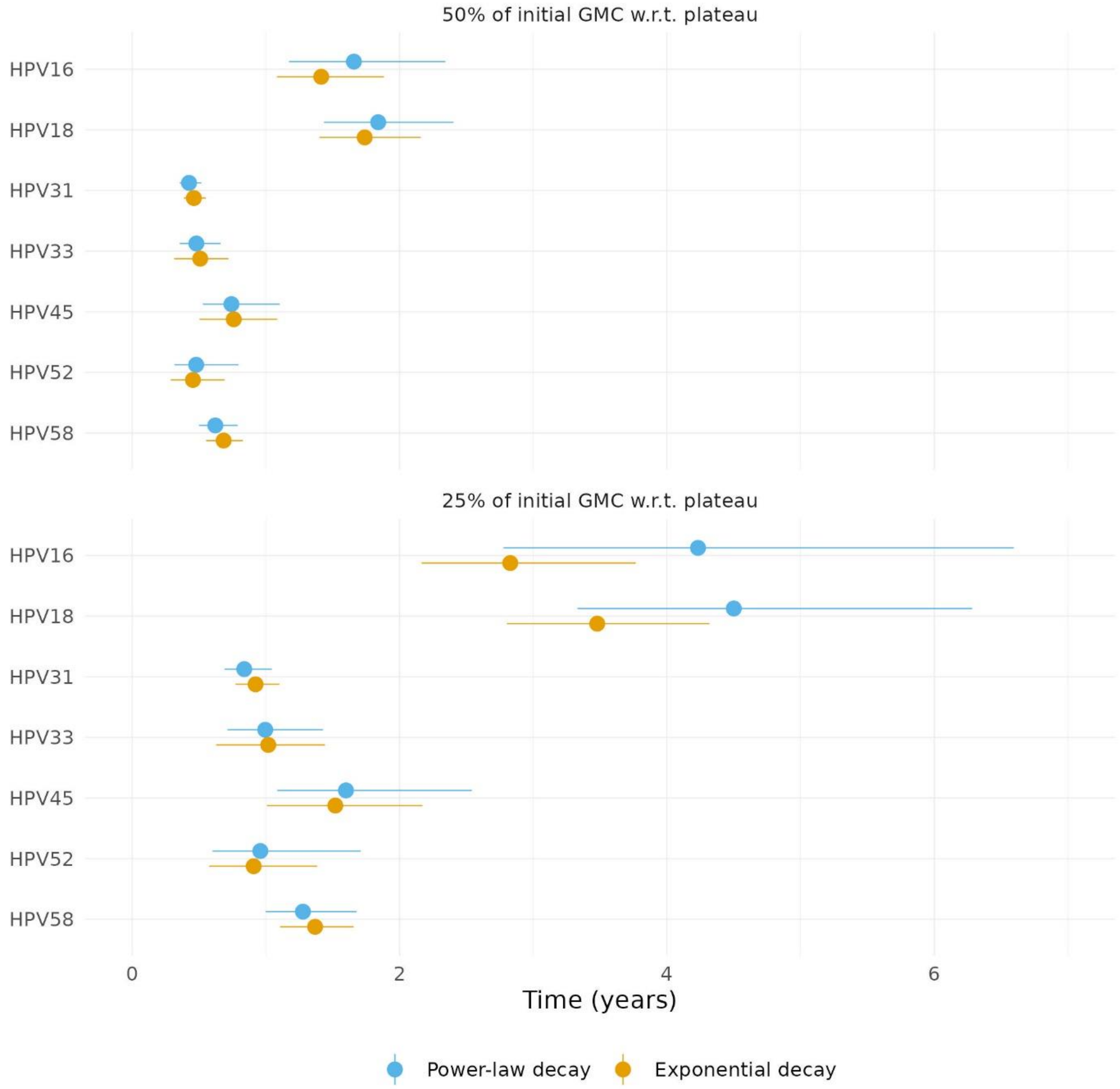

Supplementary Figure 4. Time in years to reach 50% (left) and 25% of the initial GMC with respect to the plateau GMC for each HPV type for the power-law decay model and expontential decay model.

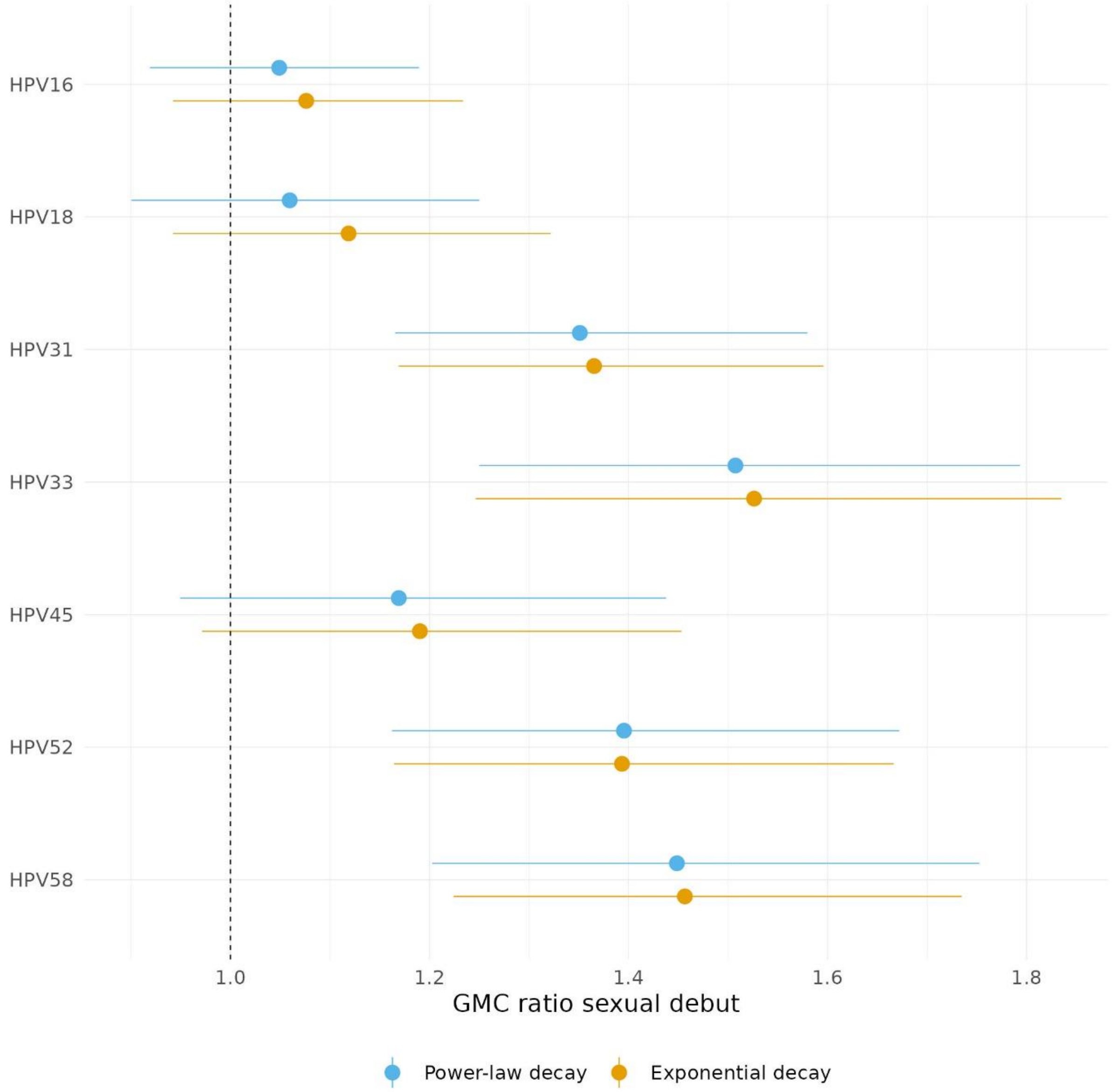

Supplementary Figure 5. Estimated GMC ratios for sexual debut and their corresponding 95% CIs for each HPV type for the power-law decay model and expontential decay model.
